# Direct and indirect evidence for pyroptosis and immune dysregulation in IVF/ICSI-ET-related early pregnancy loss

**DOI:** 10.3389/fimmu.2026.1844319

**Published:** 2026-05-22

**Authors:** Xiao Wang, Guangmou Chen, Fenghua Liu

**Affiliations:** 1 Jinan University, Guangzhou, Guangdong, China; 2 Affiliated Hospital of Guangdong Medical University, Zhanjiang, China; 3 Department of Reproductive Health and Infertility, Guangdong Province Maternity and Child Care Hospital, Guangzhou, Guangdong, China

**Keywords:** early pregnancy loss, immune imbalance, immunomodulatory therapy, IVF/ICSI-ET, NLRP3 inflammasome, pyroptosis, reproductive immunology

## Abstract

**Background:**

Early pregnancy loss(EPL) remains a major obstacle to successful *in vitro* fertilization/intracytoplasmic sperm injection-embryo transfer(IVF/ICSI-ET). Immune dysregulation at the maternal-fetal interface has been increasingly implicated in implantation failure and early miscarriage, while inflammasome-mediated pyroptosis has emerged as a potential contributor to inflammatory tissue injury. However, the direct evidence linking pyroptosis to IVF/ICSI-ET-related EPL remains limited.

**Objective:**

To critically appraise direct and indirect evidence regarding immune dysregulation and the potential role of pyroptosis in IVF/ICSI-ET-related EPL, and to define current translational limitations.

**Methods:**

A structured review of epidemiological, clinical, and mechanistic studies published between 2020 and 2025 was conducted using literature relevant to EPL, IVF/ICSI-ET, maternal-fetal immune regulation, inflammasome activation, and pyroptosis. Evidence was considered according to its direct relevance to IVF/ICSI-ET-related EPL and interpreted with attention to study design, methodological limitations, and translational applicability.

**Results:**

Established clinical predictors of EPL after IVF/ICSI-ET include maternal age, body mass index, endometrial characteristics, and embryonic or sperm-related factors. Immune abnormalities involving uterine natural killer cells, macrophage polarization, and regulatory T-cell imbalance may contribute to impaired maternal-fetal tolerance. Pyroptosis-related pathways, particularly those involving the NLRP3/ASC/Caspase-1 axis, gasdermin D cleavage, and IL-1β/IL-18 release, provide a biologically plausible framework for both physiological epithelial remodeling during implantation and inflammatory injury when dysregulated. Nevertheless, most pyroptosis-related evidence is indirect and derived from pregnancy-related complications or non-reproductive inflammatory diseases rather than from IVF/ICSI-ET-specific EPL studies.

**Conclusion:**

Immune dysregulation is likely contributes to in IVF/ICSI-ET-related EPL, whereas pyroptosis should currently be regarded as a promising but insufficiently validated mechanistic candidate. Future studies should prioritize cell-specific, temporally resolved, and clinically relevant investigations to clarify whether immune-pyroptosis interactions have diagnostic or therapeutic value in this setting.

## Introduction

With the continued advancement of assisted reproductive technologies (ART), *in vitro* fertilization/intracytoplasmic sperm injection-embryo transfer (IVF/ICSI-ET) has become a fundamental strategy for the treatment of infertility (1). Despite substantial progress in embryo culture, transfer techniques, and luteal-phase support, early pregnancy loss (EPL) remains a major obstacle to successful ART outcomes (2). EPL not only reduces clinical pregnancy and live birth rates, but also prolongs treatment, increases financial costs, and imposes considerable psychological burden on affected patients (3). Established contributors to EPL after IVF/ICSI-ET include embryonic chromosomal abnormalities, impaired endometrial receptivity, and endocrine disturbances (4). However, these factors do not fully explain the biological heterogeneity of pregnancy failure, suggesting that additional mechanisms operating at the maternal-fetal interface warrant closer investigation.

Among these mechanisms, immune regulation has attracted increasing attention because successful implantation and early placentation depend on a finely balanced uterine immune microenvironment. Under physiological conditions, coordinated interactions among uterine natural killer cells, macrophages, regulatory T cells, and trophoblasts help maintain maternal-fetal tolerance while supporting tissue remodeling and embryo development. Disruption of this balance may lead to excessive inflammatory signaling, impaired trophoblast function, and implantation failure or miscarriage (5, 6). In parallel, pyroptosis, a highly inflammatory form of programmed cell death mediated by inflammasome activation, has emerged as a biologically plausible contributor to reproductive tissue injury. In particular, activation of the NLRP3 inflammasome, recruitment of apoptosis-associated speck-like protein containing a CARD (ASC), and subsequent caspase-1 activation promote gasdermin D cleavage and the maturation and release of interleukin-1β and interleukin-18, thereby amplifying local inflammation and potentially reshaping the uterine immune microenvironment (7, 8). Nevertheless, although these pathways are increasingly discussed in pregnancy-related disorders, direct mechanistic and clinical evidence specifically linking pyroptosis to IVF/ICSI-ET-related EPL remains limited (8, 9).

Accordingly, the aim of this review is not to presume pyroptosis as an established central mechanism in IVF/ICSI-ET-related EPL, but to critically examine the current evidence regarding immune dysregulation, inflammasome activation, and pyroptosis in this setting. We first outline the clinical and immunological context of EPL after IVF/ICSI-ET, then summarize the molecular basis of pyroptosis with particular attention to the NLRP3/ASC/Caspase-1 axis and its downstream inflammatory mediators. We further distinguish between direct evidence from IVF/ICSI-ET-related EPL and indirect evidence derived from other pregnancy-related or non-reproductive inflammatory conditions, with emphasis on their strengths and limitations. By doing so, this review aims to provide a cautious mechanistic framework for understanding how immune imbalance and pyroptosis may interact at the maternal-fetal interface, while also identifying key gaps that must be addressed before meaningful diagnostic or therapeutic translation can be justified.

## Methods

### Literature search strategy and review approach

This article was designed as a structured critical review rather than a formal systematic review, with the aim of evaluating current evidence on immune dysregulation and the potential role of pyroptosis in EPL following IVF/ICSI-ET. To emphasize contemporary evidence with greater potential clinical and translational relevance, the literature search primarily focused on studies published between January 2020 and December 2025. Earlier landmark articles were selectively included when necessary to provide essential biological background on maternal-fetal immune regulation, inflammasome activation, and pyroptosis-related signaling pathways.

A structured literature search was conducted in PubMed, Web of Science, Embase, and Scopus. The search strategy combined controlled vocabulary and free-text terms related to the following concepts: “early pregnancy loss,” “miscarriage,” “IVF,” “ICSI,” “embryo transfer,” “maternal-fetal interface,” “immune dysregulation,” “immune imbalance,” “inflammasome,” “pyroptosis,” “NLRP3,” “ASC,” “caspase-1,” “gasdermin D,” “IL-1β,” and “IL-18.” Reference lists of relevant original studies and review articles were also screened manually to identify additional pertinent publications.

### Eligibility criteria and evidence framework

The primary outcome of interest in this review was IVF/ICSI-ET-related EPL. Studies were considered eligible if they were relevant to one or more of the following areas: (i) clinical or translational investigations evaluating risk factors, immune alterations, inflammatory pathways, biomarkers, or pregnancy outcomes in women undergoing IVF/ICSI-ET; (ii) experimental or mechanistic studies examining inflammasome activation or pyroptosis in reproductive tissues or models relevant to early pregnancy, including endometrium, decidua, trophoblast, placenta, implantation, or miscarriage-related contexts; and (iii) selected review articles used only to provide necessary conceptual or biological background.

To improve interpretive clarity and reduce overextrapolation, the included literature was categorized *a priori* into three evidence tiers: direct evidence, pregnancy-related indirect evidence, and non-reproductive indirect evidence. Direct evidence referred to studies specifically addressing IVF/ICSI-ET-related EPL or closely matched assisted reproduction settings with EPL-related outcomes. Pregnancy-related indirect evidence referred to studies involving miscarriage, recurrent implantation failure, decidual dysfunction, placental inflammation, endometrial inflammation, or related reproductive disorders that may inform shared inflammatory mechanisms at the maternal-fetal interface but do not directly establish disease-specific relevance to IVF/ICSI-ET-related EPL. Non-reproductive indirect evidence referred to broader inflammatory, autoimmune, infectious, metabolic, neuroinflammatory, or toxicological models that were not considered disease-specific evidence for IVF/ICSI-ET-related EPL, but were included only when they contributed mechanistic plausibility, assay-related relevance, or proof-of-concept for inflammasome- or pyroptosis-related pathways. The evidence hierarchy, its permitted interpretive uses, and its boundaries are summarized in **Table 1**.

**Table 1:** Evidence hierarchy used in this review and its interpretive boundaries.

Evidence tier	Allowed use	Not allowed to support	Example study types
Direct evidence	Can be used to support conclusions specifically related to IVF/ICSI-ET-related early pregnancy loss (EPL), including disease relevance, clinical association, and cautiously framed translational implications when supported by study quality.	Should not be overextended to claim universal mechanisms across all miscarriage or reproductive disorders without considering population and outcome differences.	Clinical or translational studies specifically evaluating women undergoing IVF/ICSI-ET with EPL-related outcomes; human reproductive tissue studies directly linked to IVF/ICSI-ET pregnancy loss.
Pregnancy-related indirect evidence	Can be used to support reproductive plausibility, maternal-fetal interface relevance, and hypothesis generation regarding shared inflammatory or immune mechanisms.	Should not be used as stand-alone evidence for disease-specific causality, validated biomarkers, or treatment efficacy in IVF/ICSI-ET-related EPL.	Studies on recurrent miscarriage, recurrent implantation failure, decidual dysfunction, placental inflammation, endometrial inflammation, trophoblast injury, or related reproductive disorders.
Non-reproductive indirect evidence	Can be used only to illustrate assay feasibility, upstream inflammasome or pyroptosis mechanisms, pathway biology, or therapeutic tractability in a general sense.	Should not be used to infer causality, diagnostic validity, biomarker specificity, or therapeutic effectiveness for IVF/ICSI-ET-related EPL.	Studies from autoimmune, infectious, neuroinflammatory, metabolic, toxicological, or other non-reproductive inflammatory disease models examining NLRP3 activation, caspase-1 signaling, gasdermin cleavage, or related pathways.

Direct evidence was assigned the greatest interpretive weight in this review. Pregnancy-related indirect evidence was used to support biological relevance at the maternal-fetal interface, whereas non-reproductive indirect evidence was used only as contextual or hypothesis-generating support and was not considered disease-specific evidence for IVF/ICSI-ET-related EPL.

Reports lacking sufficient methodological detail, duplicate publications, conference abstracts without full data, editorials, and studies judged to have no meaningful relevance to maternal-fetal immune regulation, inflammasome activation, or pyroptosis-related biology were excluded.

### Study selection and data extraction

Studies were screened according to their relevance to the central review question, namely whether immune dysregulation and pyroptosis are supported as contributors to IVF/ICSI-ET-related EPL. For each included study, the following information was extracted where available: study design, population or experimental model, reproductive context, sample source, key immune- or pyroptosis-related markers, detection methods, principal findings, and major limitations. For studies evaluating predictive models or candidate diagnostic tools, reported performance indicators were recorded, including the area under the receiver operating characteristic curve (AUC), sensitivity, specificity, and, where available, calibration or external validation results. For mechanistic studies, particular attention was paid to tissue relevance, cell-type specificity, experimental controls, and whether conclusions regarding inflammasome activation or pyroptosis were supported by complementary evidence, such as caspase activation, gasdermin cleavage, cytokine maturation, or orthogonal validation approaches.

### Quality appraisal and evidence interpretation

Given the heterogeneity of the included literature, methodological quality was appraised narratively according to study type and evidence tier rather than through a single formal risk-of-bias instrument. For observational clinical studies, emphasis was placed on sample size, population heterogeneity, confounder adjustment, and clarity of outcome definition. For interventional studies, attention was directed to comparator selection, clinically meaningful endpoints, and the extent to which the proposed mechanism was directly tested rather than inferred. For mechanistic and biomarker studies, particular consideration was given to tissue relevance, model validity, experimental controls, reproducibility, and consistency across independent samples or complementary methods.

Additional caution was applied when interpreting indirect evidence, especially non-reproductive studies, because biological plausibility does not imply disease specificity or clinical translatability in IVF/ICSI-ET-related EPL. Accordingly, stronger interpretive weight was assigned to direct IVF/ICSI-ET-related evidence, whereas pregnancy-related indirect evidence was used to support reproductive plausibility and non-reproductive indirect evidence was used only as contextual or hypothesis-generating support. This interpretive framework was applied consistently throughout the review and corresponds to the evidence boundaries outlined in **Table 1**.

### Evidence synthesis

Because the included literature comprised heterogeneous clinical studies, translational investigations, mechanistic experiments, and selected background reviews, with substantial variation in study design, tissue source, model system, and outcome definition, a quantitative meta-analysis was not considered appropriate. Instead, the evidence was synthesized descriptively and critically, with explicit attention to disease specificity, level of inference, biological plausibility, and translational limitations. Throughout the review, emphasis was placed on distinguishing established observations from mechanistic extrapolation, particularly when discussing the potential role of pyroptosis in IVF/ICSI-ET-related EPL.

### Epidemiological analysis of EPL following IVF/ICSI-ET

#### Incidence and established clinical predictors of EPL after IVF/ICSI-ET

Early pregnancy loss following *in vitro* fertilization/intracytoplasmic sperm injection-embryo transfer remains an important adverse outcome in assisted reproductive technology, despite ongoing improvements in embryo culture, laboratory conditions, and transfer strategies. Recent clinical studies suggest that miscarriage rates after IVF/ICSI-ET remain substantial, often around 14% and varying across patient populations and treatment characteristics, including endometrial preparation and other clinical factors. These data indicate that, although advances in ART may improve implantation and pregnancy rates, they have not eliminated the risk of early pregnancy loss (10, 11).

Maternal age remains one of the strongest risk factors, reflecting the contribution of declining oocyte quality and increasing embryonic chromosomal abnormalities with advancing age (12). Other frequently identified factors include increased body mass index, previous miscarriage history, thin endometrium, diminished ovarian reserve, and male sperm DNA fragmentation (13, 14). Prediction models incorporating some of these variables have shown only modest discriminative ability, with reported area under the receiver operating characteristic curve values around 0.64-0.66, indicating that currently available clinical parameters alone are insufficient to fully capture the biological complexity of EPL risk after IVF/ICSI-ET (14).

#### Why epidemiological risk factors alone are not sufficient

Although epidemiological and clinical studies have identified a range of maternal, embryonic, and uterine factors associated with EPL, these variables do not fully explain why pregnancy failure occurs in some apparently favorable IVF/ICSI-ET cycles (14). For example, not all losses can be attributed to chromosomal abnormalities, endocrine insufficiency, or endometrial thickness alone, and even patients with similar baseline characteristics may experience markedly different reproductive outcomes. This heterogeneity suggests that additional mechanisms at the maternal-fetal interface, particularly those involving local immune regulation and inflammatory responses, may contribute to pregnancy loss (15, 16).

From this perspective, the epidemiology of EPL after IVF/ICSI-ET should be interpreted not only as a collection of clinical risk factors but also as an indicator of unresolved biological mechanisms. In particular, the incomplete predictive performance of current risk models highlights the need to investigate pathway-level processes that may underlie implantation failure and early embryonic loss. These considerations provide a rationale for examining maternal-fetal immune dysregulation and, more cautiously, the possible contribution of inflammasome activation and pyroptosis-related inflammatory injury in IVF/ICSI-ET-related EPL (17, 18).

### The role of immune imbalance in early pregnancy loss following IVF/ICSI-ET

#### Maternal-fetal immune homeostasis in early pregnancy

Successful implantation and early pregnancy maintenance require a tightly regulated immune microenvironment at the maternal-fetal interface, in which uNK cells, decidual macrophages, Tregs, and trophoblasts interact coordinately to support trophoblast invasion, vascular remodeling, tissue homeostasis, and maternal-fetal tolerance (19, 20). Rather than mounting a destructive immune response against the semi-allogeneic embryo, the maternal immune system establishes a controlled inflammatory-tolerogenic balance that permits placentation while limiting excessive tissue injury. This dynamic equilibrium is essential during the peri-implantation period and early gestation, when subtle disturbances in local immune signaling may have disproportionate effects on embryonic development (21).

Among the major cellular components of this microenvironment, uNK cells are particularly abundant in the endometrium during the implantation window and early pregnancy. These cells differ functionally from peripheral NK cells and are involved in spiral artery remodeling, cytokine secretion, and trophoblast support (22). Decidual macrophages also play a central role by balancing pro-inflammatory and tissue-remodeling functions, while Tregs contribute to maternal-fetal tolerance by suppressing excessive immune activation. Together, these immune populations help maintain a local environment that is permissive for implantation and early placental formation (23, 24).

#### Immune dysregulation and its association with EPL after IVF/ICSI-ET

When maternal-fetal immune homeostasis is disturbed, the risk of implantation failure and EPL may increase. Clinical and translational studies have reported alterations in immune cell composition and function in women with adverse reproductive outcomes, including recurrent implantation failure and early miscarriage. Abnormalities in uNK cell number or activity, disruption of macrophage polarization, reduced Treg-mediated tolerance, and altered helper T-cell responses have all been associated with a pro-inflammatory uterine milieu that may impair trophoblast invasion and embryonic development (22, 25).

In particular, macrophage imbalance is considered an important feature of reproductive immune dysfunction. Under normal conditions, decidual macrophages exhibit functional plasticity, with M1-like phenotypes contributing to controlled inflammatory responses and M2-like phenotypes supporting tissue repair, immune tolerance, and placental development (26). A shift toward excessive M1-associated inflammation may enhance cytokine production, oxidative stress, and local tissue injury, thereby creating conditions unfavorable for pregnancy maintenance. Similarly, dysregulated uNK cell function may interfere with endometrial receptivity and vascular adaptation, while reduced Treg activity may weaken maternal tolerance to fetal antigens (27).

Humoral immune abnormalities may further contribute to this inflammatory imbalance. For example, antinuclear antibody (ANA) positivity has been associated with poorer IVF/ICSI outcomes and a higher risk of early miscarriage in some patient populations (28, 29). Although such findings do not establish direct causality, they support the concept that immune activation and loss of tolerance may participate in the pathophysiology of EPL after assisted reproduction.

#### Potential links between immune dysregulation and inflammasome activation

Although immune dysregulation and pyroptosis are often discussed separately in the current literature, there are plausible biological connections between these processes. A persistently pro-inflammatory maternal-fetal microenvironment may promote the accumulation of cytokines, reactive oxygen species, and damage-associated molecular patterns (DAMPs), all of which are known to facilitate inflammasome activation. In this context, excessive inflammatory signaling could prime the NLRP3 inflammasome pathway, leading to caspase-1 activation, gasdermin D cleavage, and the maturation and release of interleukin-1β and interleukin-18 (30, 31).

**Figure 1:**
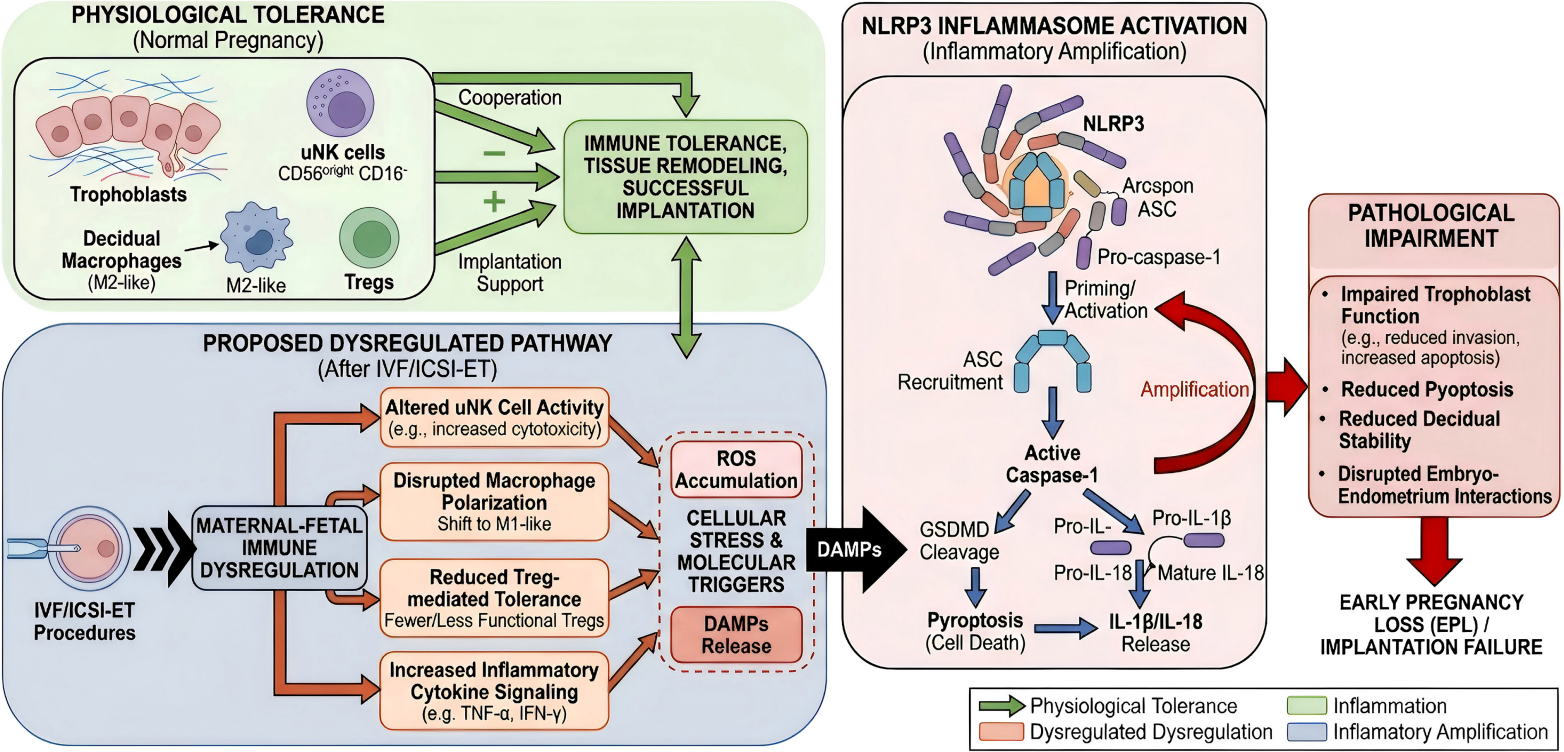
Proposed framework linking maternal-fetal immune dysregulation, inflammasome activation, and early pregnancy loss after IVF/ICSI-ET. This figure summarizes a proposed mechanistic framework, rather than a conclusively established pathway, linking maternal-fetal immune dysregulation to EPL after IVF/ICSI-ET. Under physiological conditions, uterine natural killer cells, decidual macrophages, regulatory T cells (Tregs), and trophoblasts cooperate to maintain immune tolerance, tissue remodeling, and implantation support. Under dysregulated conditions, altered uNK cell activity, disrupted macrophage polarization, reduced Treg-mediated tolerance, and increased inflammatory cytokine signaling may promote reactive oxygen species accumulation and release of damage-associated molecular patterns (DAMPs). These events may prime or activate the NLRP3 inflammasome, leading to ASC recruitment, caspase-1 activation, gasdermin D (GSDMD) cleavage, and release of IL-1β and IL-18. The resulting inflammatory amplification may impair trophoblast function, decidual stability, and embryo-endometrium interactions, thereby increasing the risk of implantation failure or EPL. Because direct mechanistic evidence in IVF/ICSI-ET-related EPL remains limited, this framework should be interpreted as biologically plausible but still requiring validation.

Such events may amplify local inflammatory injury and further disrupt the uterine.

microenvironment, thereby creating a self-reinforcing cycle between immune imbalance and tissue damage. At the maternal-fetal interface, this process could theoretically affect trophoblasts, decidual immune cells, or endometrial stromal cells, ultimately compromising implantation stability and early embryonic survival (32). However, direct evidence demonstrating this immune-pyroptosis interaction specifically in IVF/ICSI-ET-related EPL remains limited. Therefore, current data support a mechanistically plausible link rather than a conclusively established pathway. **Figure 1** summarizes the proposed framework linking maternal-fetal immune dysregulation, inflammasome activation, and EPL after IVF/ICSI-ET. The main immune abnormalities relevant to IVF/ICSI-ET-related EPL and adjacent reproductive disorders are summarized in **Table 2**.

**Table 2:** Immune abnormalities relevant to IVF/ICSI-ET-related EPL and adjacent reproductive disorders: biological roles, reported alterations, and mechanistic implications.

Immune component	Physiological role in early pregnancy	Reported alteration in EPL or related reproductive disorders	Potential mechanistic implication	Evidence level
uNK cells	Support implantation, vascular remodeling, trophoblast interaction	Abnormal number or altered functional activity	Impaired endometrial receptivity and local inflammatory imbalance	Pregnancy-related indirect to moderate
Decidual macrophages	Balance inflammation, tissue repair, and tolerance	Shift toward excessive M1-like inflammatory polarization	Increased cytokine production, oxidative stress, and tissue injury	Pregnancy-related indirect to moderate
Regulatory T cells (Tregs)	Maintain maternal-fetal tolerance	Reduced suppressive activity or relative deficiency	Loss of tolerance and enhanced inflammatory activation	Pregnancy-related indirect to moderate
Helper T-cell subsets	Coordinate adaptive immune balance	Altered Th-related signaling, including pro-inflammatory skewing	Amplified local immune dysregulation	Limited to indirect
ANA and related immune activation	Reflect systemic immune dysregulation	Positive association with poorer IVF/ICSI outcomes in some cohorts	Suggestive of immune intolerance rather than direct mechanism	Clinical association only

uNK, uterine natural killer; EPL, early pregnancy loss; ANA, antinuclear antibody. This table summarizes immune abnormalities relevant to maternal-fetal tolerance and does not imply that all listed alterations are specific to IVF/ICSI-ET-related EPL.

### Mechanisms of the inflammatory pyroptosis pathway in early pregnancy loss after IVF/ICSI-ET

#### Molecular basis of inflammasome-mediated pyroptosis

Pyroptosis is a lytic and pro-inflammatory form of programmed cell death that is typically triggered by inflammasome activation. Among the currently known inflammasomes, the NLRP3 inflammasome is the most extensively studied and consists of the sensor protein NLRP3, the adaptor apoptosis-associated speck-like protein containing a CARD (ASC), and pro-caspase-1. Upon exposure to pathogen-associated molecular patterns (PAMPs), damage-associated molecular patterns (DAMPs), oxidative stress, or other inflammatory stimuli, NLRP3 undergoes activation and recruits ASC, which promotes the cleavage and activation of caspase-1. Activated caspase-1 subsequently cleaves gasdermin D (GSDMD), releasing its N-terminal fragment to form membrane pores, thereby inducing cell swelling, membrane rupture, and inflammatory cell death. Caspase-1 also processes the pro-inflammatory cytokines interleukin-1β and interleukin-18 into their mature forms, further amplifying local inflammatory responses (33, 34).

In addition to the canonical NLRP3/ASC/caspase-1 pathway, non-canonical pyroptosis may be initiated through caspase-4 and caspase-5 in humans, or caspase-11 in mice, particularly in response to intracellular lipopolysaccharide. Although the upstream triggers differ, both canonical and non-canonical pathways converge on GSDMD cleavage and inflammatory membrane pore formation. In biological systems characterized by persistent inflammatory stimulation, these pathways may interact with oxidative stress, mitochondrial dysfunction, and cytokine amplification, thereby contributing to tissue injury beyond host defense alone. At the maternal-fetal interface, such processes are biologically relevant because excessive inflammatory injury could impair trophoblast function, decidual integrity, and implantation stability (34, 35).

#### Current evidence relevant to pregnancy and reproductive tissues

Interest in pyroptosis has increased in reproductive medicine because inflammasome activation has been implicated in several pregnancy-related disorders and inflammatory conditions affecting reproductive tissues. Studies in miscarriage-related or pregnancy-associated settings have suggested that aberrant activation of the NLRP3 inflammasome may contribute to inflammatory injury through increased IL-1β and IL-18 release, oxidative stress, and disruption of local immune homeostasis (36, 37).

In particular, inflammatory conditions involving the endometrium or maternal-fetal interface provide a biologically plausible context in which pyroptosis may influence implantation and early embryonic development (38).However, the current evidence base is uneven and should be interpreted with caution. Direct studies specifically examining pyroptosis in IVF/ICSI-ET-related early pregnancy loss remain limited. Much of the available literature is instead derived from adjacent reproductive contexts, such as endometrial inflammation, recurrent miscarriage, placental dysfunction, or broader pregnancy-related immune disorders. These studies support the possibility that inflammasome-mediated inflammatory injury may be relevant to early pregnancy failure, but they do not yet establish pyroptosis as a confirmed central mechanism in IVF/ICSI-ET-related EPL (39).

Oxidative stress is one potential link between reproductive immune disturbance and pyroptosis. Reactive oxygen species can promote NLRP3 inflammasome activation, and oxidative stress-related signaling has been implicated in tissue injury across multiple inflammatory models (40). In the setting of IVF/ICSI-ET, several ART-related procedures may provide specific biological contexts in which inflammatory priming and pyroptosis-related signaling warrant investigation. Controlled ovarian stimulation may expose the endometrium to supraphysiological hormonal conditions and alter vascular permeability, oxidative stress, and immune readiness. Luteal-phase dynamics and luteal support may further influence decidualization, cytokine production, and embryo-endometrial synchronization. In addition, embryo culture, micromanipulation, cryopreservation, thawing, and embryo transfer timing may modify embryonic stress signals or the temporal coordination between embryo development and endometrial receptivity. Endometrial preparation protocols in frozen embryo transfer cycles may also shape local immune and inflammatory states (41, 42). However, these considerations should be interpreted as IVF/ICSI-ET-specific mechanistic contexts rather than direct evidence that ART procedures induce clinically meaningful pyroptosis-mediated EPL. Accordingly, oxidative stress should currently be regarded as a plausible upstream contributor rather than definitive proof of pyroptosis-driven EPL in IVF/ICSI-ET (32, 43).

### Physiological pyroptosis during embryo implantation: the HOXA10-NLRP3 regulatory axis

Although pyroptosis is frequently discussed as a pathological mechanism of inflammatory tissue injury, it may also participate in physiological implantation when it is transient, spatially restricted, and tightly regulated. Embryo implantation requires local remodeling and partial clearance of the luminal epithelium to permit embryo attachment and invasion. Recent mechanistic evidence from Ashary et al. showed that loss of HOXA10 activates NLRP3-dependent epithelial plasticity and pyroptosis in the endometrium during embryo implantation (44). HOXA10 normally helps preserve the epithelial identity of luminal epithelial cells, whereas local downregulation of HOXA10 during implantation permits upregulation of NLRP3. NLRP3 then acts as a downstream effector promoting epithelial plasticity, partial epithelial-to-mesenchymal transition, inflammasome activation, and GSDMD-mediated pyroptosis in luminal epithelial cells. This controlled pyroptotic process contributes to localized epithelial clearance at the implantation site and facilitates embryo invasion.

This evidence indicates that pyroptosis should not be viewed exclusively as a detrimental process in early pregnancy. Instead, pyroptosis at the maternal-fetal interface may have a biphasic and context-dependent role. Insufficient pyroptosis may impair luminal epithelial cell clearance and hinder embryo implantation, whereas physiological pyroptosis, when transient and spatially restricted, may support epithelial remodeling and embryo invasion. Conversely, excessive or spatially uncontrolled pyroptosis may destroy an overly broad epithelial area, amplify inflammatory cytokine release, and disrupt embryo-endometrial communication, thereby increasing the risk of implantation failure or EPL. Therefore, the key pathological issue may not be the presence of pyroptosis itself, but the disruption of its physiological regulation, including inappropriate timing, excessive magnitude, prolonged activation, or loss of spatial restriction. In the context of IVF/ICSI-ET, this concept provides a useful mechanistic framework for future studies examining whether controlled ovarian stimulation, luteal-phase dynamics, embryo manipulation, or endometrial preparation may disturb the physiological balance of inflammasome activation and epithelial remodeling.

### Interpretive framework for IVF/ICSI-ET-related EPL

Taken together, existing data suggest that pyroptosis provides a mechanistically plausible framework for understanding inflammatory injury in early pregnancy, especially when considered alongside immune dysregulation at the maternal-fetal interface. A hypothetical sequence may involve inflammatory priming, ROS accumulation, and DAMP release, followed by NLRP3 inflammasome activation, caspase-1 cleavage, GSDMD-mediated membrane pore formation, and enhanced secretion of IL-1β and IL-18. This cascade could theoretically damage trophoblasts, decidual immune cells, or endometrial stromal cells, thereby amplifying local inflammation and compromising implantation or early embryonic survival (45, 46).

In this framework, the pathological relevance of pyroptosis may depend not only on its activation, but also on its timing, intensity, spatial restriction, and cellular context. Thus, both insufficient pyroptotic clearance and excessive pyroptotic injury may theoretically impair implantation and early pregnancy maintenance.

Conceptually, pyroptosis in IVF/ICSI-ET-related EPL may be positioned in three non-mutually exclusive ways. First, it may act as a primary driver in selected cases if inflammasome activation directly disrupts trophoblast function, decidual integrity, or epithelial remodeling before pregnancy failure becomes clinically apparent. Second, pyroptosis may function as an inflammatory amplifier, in which pre-existing immune imbalance, oxidative stress, or ART-related inflammatory priming activates the NLRP3/ASC/caspase-1/GSDMD pathway, leading to IL-1β/IL-18 release, DAMP accumulation, and further immune activation (30, 32–34). Third, pyroptosis may occur as a secondary consequence of upstream embryonic, endocrine, vascular, or immune disturbances. Based on the current evidence, pyroptosis in IVF/ICSI-ET-related EPL is best regarded as a context-dependent and insufficiently validated mechanism, with stronger present support for its role as an inflammatory amplifier or downstream consequence than as a universally established primary cause (8, 9, 32, 39).

**Figure 2:**
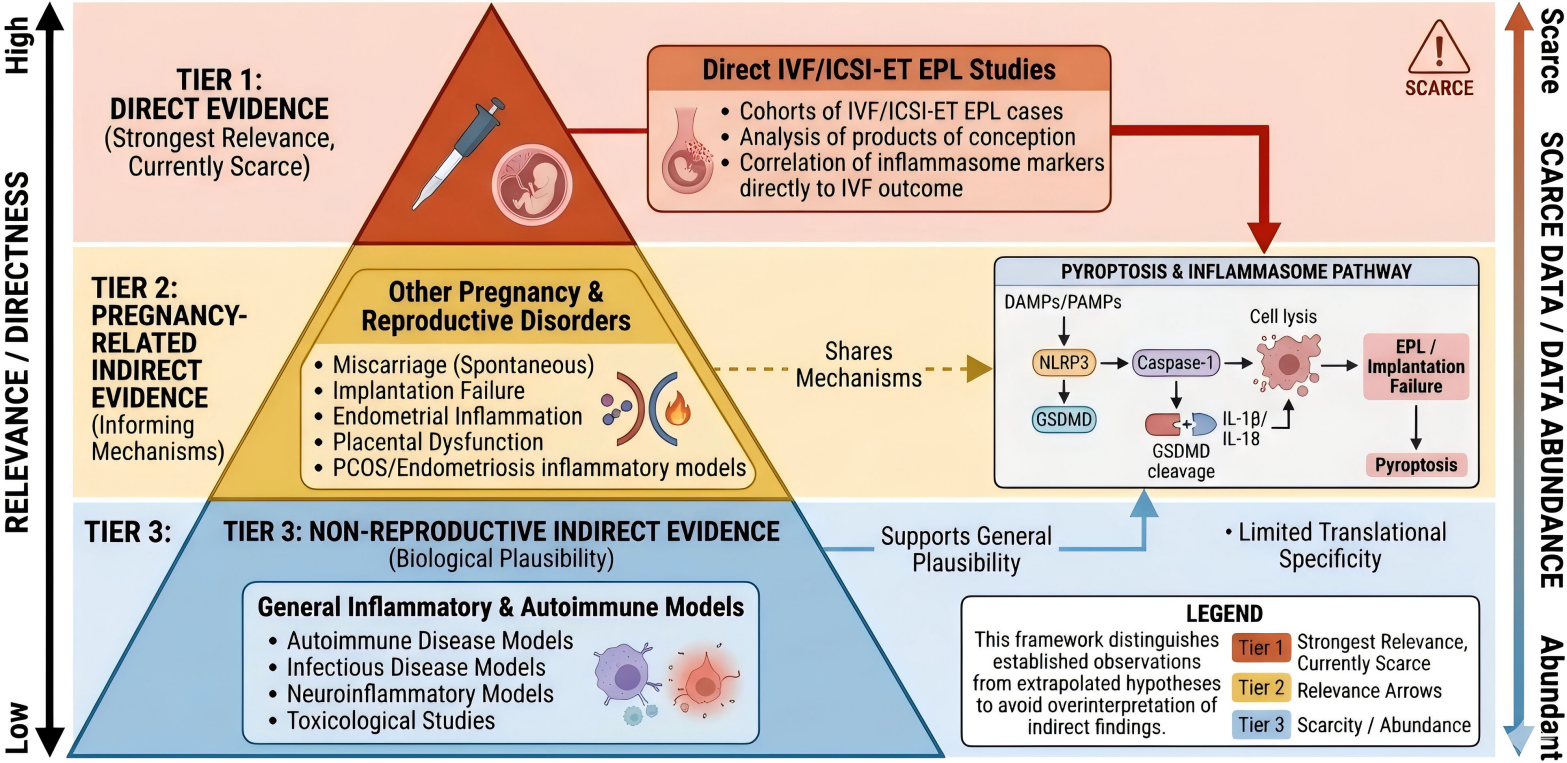
Levels of evidence supporting the role of pyroptosis in IVF/ICSI-ET-related early pregnancy loss. This figure illustrates the evidence hierarchy discussed in the review. The strongest relevance is assigned to direct evidence from studies specifically addressing IVF/ICSI-ET-related EPL, although such studies are currently scarce. A second tier includes pregnancy-related indirect evidence, such as studies on miscarriage, implantation failure, endometrial inflammation, placental dysfunction, or other reproductive disorders that may inform maternal-fetal inflammatory mechanisms. A third tier includes non-reproductive indirect evidence from autoimmune, infectious, neuroinflammatory, or toxicological models, which may support biological plausibility of inflammasome activation and pyroptosis but have limited translational specificity for early pregnancy. This evidence hierarchy is intended to distinguish established observations from extrapolated hypotheses and to avoid overinterpretation of indirect findings.

Importantly, this proposed framework should not be interpreted as conclusive evidence. At present, the literature supports biological plausibility more strongly than direct clinical validation. Therefore, in the context of IVF/ICSI-ET-related EPL, pyroptosis is better regarded as an emerging mechanistic candidate rather than an established pathogenic pathway. Future studies should focus on cell type-specific analyzes, temporally resolved tissue sampling, and clinically relevant human or translational models to determine whether inflammasome-mediated pyroptosis has a causal, diagnostic, or therapeutic role in this setting. **Figure 2** illustrates the levels of evidence supporting the role of pyroptosis in IVF/ICSI-ET-related EPL. The evidence categories, their relevance to IVF/ICSI-ET-related EPL, and their major limitations are summarized in **Table 3**.

**Table 3:** Evidence map for pyroptosis-related mechanisms relevant to IVF/ICSI-ET-related EPL.

Evidence category	Clinical or experimental context	Main pyroptosis-related implication	Relevance to IVF/ICSI-ET-related EPL	Major limitation
Direct evidence	Studies specifically addressing IVF/ICSI-ET-related EPL	Currently scarce	Highest relevance	Insufficient quantity and validation
Pregnancy-related indirect evidence	Miscarriage, implantation failure, endometrial inflammation, placental disorders	Suggests inflammasome activation may contribute to inflammatory injury at the maternal-fetal interface	Moderate relevance	Often not IVF/ICSI-ET-specific
Reproductive mechanistic evidence	Endometrium, decidua, trophoblast, or placental models	Supports biological plausibility of NLRP3/ASC/Caspase-1 signaling and downstream inflammation	Moderate relevance	Variable models and limited clinical correlation
Non-reproductive indirect evidence	Autoimmune, infectious, neuroinflammatory, or toxicological diseases	Demonstrates assay feasibility and broad inflammatory roles of pyroptosis	Low disease specificity	Limited translational relevance to early pregnancy
Oxidative stress-related observations	ROS-associated inflammatory models	Suggests ROS may act upstream of inflammasome activation	Hypothesis-generating	Does not establish causality in IVF/ICSI-ET-related EPL

Evidence categories are arranged according to disease specificity, not publication volume. The presence of indirect evidence should not be interpreted as proof of a central pathogenic role in IVF/ICSI-ET-related EPL.

### Potential pyroptosis-related biomarker candidates in IVF/ICSI-ET-related EPL

Pyroptosis-related molecules have attracted interest as potential biomarker candidates for inflammatory injury in early pregnancy loss following *in vitro* fertilization/intracytoplasmic sperm injection-embryo transfer. From a mechanistic perspective, components of the inflammasome pathway, including NLRP3, ASC (PYCARD), caspase-1, gasdermin D, interleukin-1β, and interleukin-18, are biologically plausible candidates because they reflect different stages of inflammasome activation, pyroptotic execution, and downstream inflammatory amplification. If validated in clinically relevant reproductive tissues or accessible biological samples, such markers may help characterize inflammatory states at the maternal-fetal interface (47, 48).

However, the current evidence supporting the diagnostic value of these markers in IVF/ICSI-ET-related EPL remains limited. Most available studies do not directly assess these molecules in patients with IVF/ICSI-ET-related miscarriage, and much of the supporting literature is derived from adjacent pregnancy disorders or from non-reproductive inflammatory diseases (49, 50). Although such studies may demonstrate assay feasibility or biological plausibility, they do not establish disease specificity or clinical utility in the setting of assisted reproduction (51). Therefore, pyroptosis-related molecules should currently be regarded as exploratory biomarker candidates rather than validated diagnostic markers for IVF/ICSI-ET-related EPL.

An additional challenge lies in the choice of sample source. Endometrial, decidual, trophoblast-derived, and peripheral blood samples differ substantially in biological relevance and clinical accessibility (52, 53). Tissue-based markers may more directly reflect local inflammatory processes at the maternal-fetal interface (52), whereas circulating biomarkers are generally more feasible for routine assessment but may provide lower tissue specificity P]. In addition, inflammasome-related mediators such as IL-1β and IL-18 are not specific to reproductive disorders and can be elevated across diverse inflammatory conditions (54, 55), limiting their standalone discriminatory value.

Accordingly, future biomarker studies should prioritize clinically relevant sample types, standardized detection methods, and validation in well-defined IVF/ICSI-ET cohorts. Comparative analyzes against established reproductive predictors, including clinical risk factors, ultrasound findings, and embryo-related parameters, will also be necessary to determine whether pyroptosis-related markers provide incremental diagnostic value rather than merely reflecting nonspecific inflammation (56, 57).

Clinically, future biomarker studies may be most informative in well-defined IVF/ICSI-ET subgroups rather than in unselected ART populations. Potential priority scenarios include unexplained EPL after embryo transfer, EPL after euploid embryo transfer, recurrent implantation failure with suspected immune imbalance, repeated biochemical pregnancy loss, thin or poorly responsive endometrium, frozen embryo transfer cycles using different endometrial preparation protocols, and patients with systemic or local inflammatory features. In these settings, pyroptosis-related markers should be evaluated alongside established clinical, embryonic, endometrial, and immune predictors to determine whether they provide incremental predictive value rather than merely reflecting nonspecific inflammation (56, 57).

### Diagnostic approaches in IVF/ICSI-ET-related EPL: established tools and exploratory candidates

Current diagnostic evaluation of EPL following IVF/ICSI-ET still relies primarily on established clinical and reproductive indicators rather than on validated pyroptosis-specific assays. Commonly used approaches include assessment of maternal age, body mass index, reproductive history, ovarian reserve, endometrial characteristics, ultrasonographic monitoring, and embryo-related parameters such as chromosomal status and developmental quality. In addition, selected immune-related assessments, including antinuclear antibodies, thyroid-related autoantibodies, and endometrial immune profiling, may provide supportive information in specific clinical contexts. These tools are more directly connected to current reproductive practice than pyroptosis-related testing and therefore remain the diagnostic foundation in this field (58, 59).

Several predictive models integrating clinical variables have been developed for EPL risk stratification after IVF/ICSI-ET. However, their reported performance has been variable and often only moderate, indicating limited discriminative power when used alone. This incomplete predictive ability highlights the need to explore additional pathway-level markers, including immune and inflammatory indicators, while also recognizing that new biomarkers must demonstrate clinically meaningful improvement beyond existing models (60, 61).

Within this context, pyroptosis-related diagnostic approaches should be viewed as exploratory rather than established. Potential methods include quantification of inflammasome-associated transcripts or proteins, measurement of IL-1β and IL-18 release, and detection of activated caspase-1 or GSDMD cleavage in reproductive tissues or blood-derived samples (62). These methods may be useful for mechanistic investigation and hypothesis generation, particularly in translational studies focusing on the maternal-fetal interface. Nevertheless, substantial barriers remain before such assays can be incorporated into clinical diagnosis, including uncertain tissue specificity, lack of assay standardization and clinically validated thresholds, limited access to reproductive tissue samples, and the current lack of prospective validation in well-defined IVF/ICSI-ET-related EPL cohorts (62, 63).

Integrated diagnostic strategies may ultimately prove more useful than any single biomarker alone. A rational future approach would combine established clinical predictors with immune profiling and carefully validated inflammatory or pyroptosis-related markers to improve biological resolution and risk stratification. At present, however, there is insufficient evidence to support routine clinical application of pyroptosis-based diagnostic testing in IVF/ICSI-ET-related EPL. Accordingly, these techniques should be interpreted as investigational tools whose translational value remains to be determined. Current diagnostic approaches and exploratory inflammatory candidates in IVF/ICSI-ET-related EPL are summarized in **Table 4**.

**Table 4:** Diagnostic approaches in IVF/ICSI-ET-related EPL: established tools and exploratory inflammatory candidates.

Diagnostic category	Representative examples	Sample source	Current status	Main limitation
Clinical risk assessment	Age, BMI, prior miscarriage, ovarian reserve, endometrial thickness	Clinical records	Established	Incomplete biological resolution
Embryo-related assessment	Embryo morphology, chromosomal evaluation	Embryo/laboratory data	Established	Does not capture maternal-fetal inflammatory mechanisms
Immune profiling	uNK cells, Tregs, macrophage-related assessment	Endometrial biopsy or peripheral blood	Supportive in selected settings	Limited standardization and tissue accessibility
Autoantibody testing	ANA, thyroid-related antibodies	Serum	Adjunctive only	Limited specificity for EPL
Candidate inflammatory markers	IL-1β, IL-18, NLRP3, CASP1, PYCARD, GSDMD	Tissue or blood-derived samples	Exploratory	Uncertain specificity, thresholds, and validation
Integrated predictive models	Multivariable nomograms with clinical variables	Clinical and laboratory data	Modest discriminative performance	AUC generally moderate; limited external validation

Pyroptosis-related molecules are currently exploratory candidates and should not be interpreted as validated diagnostic biomarkers for IVF/ICSI-ET-related EPL.

### Therapeutic implications in IVF/ICSI-ET-related EPL: evidence level, mechanistic relevance, and current limitations

#### Current therapeutic approaches relevant to EPL

Following IVF/ICSI-ET primarily target established clinical risk factors or putative immune dysfunction rather than validated pyroptosis-specific mechanisms. Accordingly, therapeutic implications in this field should be interpreted according to the strength of available evidence and the degree to which a given intervention is directly linked to maternal-fetal immune regulation or inflammasome-mediated inflammatory injury. At present, most interventions discussed in the literature are better supported as modulators of reproductive or immune status than as confirmed regulators of pyroptosis in IVF/ICSI-ET-related EPL (36, 64).

#### Immune-modulating interventions with clinical but limited mechanistic specificity

Among immune-directed interventions, intravenous immunoglobulin (IVIg) has been investigated in selected high-risk reproductive populations, particularly in women with unexplained recurrent implantation failure or suspected immune imbalance. Retrospective data suggest that IVIg may improve live birth outcomes in some subgroups; however, these findings are derived from limited clinical settings and remain subject to selection bias and uncertainty regarding patient stratification. More importantly, there is currently no direct evidence demonstrating that IVIg reduces EPL after IVF/ICSI-ET through modulation of inflammasome activation or pyroptosis pathways. Thus, IVIg should be regarded as a clinically explored immunomodulatory therapy rather than as a pyroptosis-targeted intervention (65, 66).

Other immunomodulatory or adjunctive treatments require similarly cautious interpretation. Prednisone has not shown clear benefit in improving live birth rates in patients with recurrent implantation failure, despite its anti-inflammatory rationale (67). Dehydroepiandrosterone (DHEA) has been associated with improved endometrial thickness and pregnancy-related outcomes in some studies of diminished ovarian reserve or poor ovarian response, but its effects on live birth and miscarriage remain inconsistent and heterogeneous (68). Granulocyte colony-stimulating factor (G-CSF) and platelet-rich plasma (PRP) have also attracted interest as adjunctive reproductive therapies, yet their evidence base is limited by small sample size, variable protocols, and uncertain relevance to inflammasome biology (69, 70). Overall, these treatments may influence immune balance, endometrial receptivity, or reproductive performance, but their relationship to pyroptosis regulation in IVF/ICSI-ET-related EPL remains unproven.

#### Pyroptosis-related therapeutic concepts remain preclinical and indirect

Inflammasome- or pyroptosis-related therapeutic strategies are conceptually appealing because they may interrupt inflammatory amplification at the maternal-fetal interface. Potential targets include NLRP3 inflammasome activation, caspase-1 signaling, gasdermin D-mediated membrane pore formation, and upstream triggers such as oxidative stress (71). However, evidence supporting these approaches in the specific context of IVF/ICSI-ET-related EPL is currently indirect and largely preclinical. Most studies cited in support of pyroptosis-targeted therapy have been conducted in non-reproductive inflammatory disease models, where inhibition of NLRP3 signaling reduces tissue injury and inflammatory cytokine release S,T]. Although these findings support biological plausibility, they cannot be assumed to translate directly to early pregnancy physiology, implantation biology, or assisted reproduction outcomes (72).

Future therapeutic research should focus on biologically defined scenarios in which inflammasome activation or pyroptosis-related signaling is demonstrably abnormal, rather than applying pyroptosis-targeted strategies broadly to all IVF/ICSI-ET patients. Potential research directions include evaluating whether modulation of upstream inflammatory priming, oxidative stress, NLRP3 activation, caspase-1 signaling, or GSDMD-mediated pore formation can restore maternal-fetal immune balance in carefully selected models or patient subgroups (71, 72). Importantly, such interventions must be evaluated with attention to timing and physiological context, because controlled inflammatory responses and epithelial remodeling are required for normal implantation. Therefore, the therapeutic goal should not be complete suppression of pyroptosis-related pathways, but restoration of an appropriate inflammatory balance that supports implantation while preventing excessive tissue injury.

For this reason, pyroptosis-targeted interventions should currently be described as mechanistic hypotheses rather than clinically actionable therapies in IVF/ICSI-ET-related EPL. Before such strategies can be considered translationally relevant, studies must clarify which maternal-fetal interface cell types undergo pyroptosis, when inflammasome activation occurs during implantation or early gestation, and whether suppressing these pathways improves clinically meaningful reproductive outcomes without compromising physiological immune adaptation.

#### Supportive and preventive measures should be distinguished from mechanism-directed therapy

Some supportive interventions, including thyroid function optimization (73), hormonal support (74), and selected lifestyle measures (75), may contribute to improved reproductive outcomes in appropriately selected patients. However, these approaches should not be overinterpreted as evidence for pyroptosis-targeted treatment. Their effects are more plausibly mediated through broader endocrine, metabolic, or inflammatory pathways rather than through specific modulation of the NLRP3/ASC/Caspase-1 axis (73–75). Therefore, while such measures may remain clinically relevant within individualized reproductive care, they should be distinguished from therapies that are explicitly intended to address immune-pyroptosis interactions.

#### Toward rational integrated therapeutic strategies

A more realistic near-term approach may be to develop integrated treatment frameworks that combine established reproductive management with biologically informed immune assessment, rather than prematurely proposing multimodal regimens that presume confirmed pyroptosis involvement (76, 77). In principle, future therapeutic strategies could incorporate patient stratification based on immune phenotype, endometrial environment, and validated inflammatory markers, with targeted interventions tailored to biologically defined subgroups (76, 77). However, there is insufficient evidence to recommend combined immune-pyroptosis-directed treatment protocols for routine use in IVF/ICSI-ET-related EPL (77).

Taken together, the therapeutic literature suggests that immune-modulating interventions may have selective clinical utility in some reproductive settings (78), whereas pyroptosis-targeted approaches remain exploratory (7). The major priority for future research is not the immediate clinical adoption of such treatments, but the generation of direct mechanistic and translational evidence linking immune dysregulation, inflammasome activation, and EPL after IVF/ICSI-ET (8). Until then, therapeutic claims in this area should remain cautious, evidence-graded, and explicitly limited by the current lack of IVF/ICSI-ET-specific validation. The therapeutic implications, mechanistic relevance, and major limitations of current interventions are summarized in **Table 5**.

**Table 5:** Therapeutic implications in IVF/ICSI-ET-related EPL: evidence level, mechanistic relevance, and major limitations.

Intervention group	Representative examples	Main reported effect	Relationship to pyroptosis	Evidence level/limitation
Immune-modulating interventions	IVIg	Possible benefit in selected high-risk reproductive populations	No direct evidence of pyroptosis modulation	Retrospective or subgroup-based evidence; patient selection uncertain
Glucocorticoids	Prednisone	No clear improvement in live birth in RCT settings	Indirect anti-inflammatory rationale only	Negative or limited clinical evidence
Ovarian/endometrial adjuncts	DHEA, G-CSF, PRP	Variable effects on endometrial or pregnancy-related outcomes	Mechanistic link to pyroptosis not established	Heterogeneous protocols and outcomes
Supportive reproductive management	Hormonal support, thyroid optimization, lifestyle measures	May improve overall reproductive conditions in selected patients	Not pyroptosis-specific	Broad supportive relevance only
Experimental inflammasome-targeted concepts	NLRP3 inhibition, caspase-1-related modulation, anti-oxidative strategies	Preclinical reduction of inflammatory injury in non-reproductive models	Conceptually relevant but unvalidated in IVF/ICSI-ET-related EPL	Indirect and preclinical evidence only
Integrated future strategies	Biologically stratified multimodal approaches	Hypothetical potential	Requires validated mechanistic framework first	Not ready for routine clinical use

This table distinguishes clinically used reproductive interventions from exploratory pyroptosis-related therapeutic concepts. The latter remain preclinical and should not be interpreted as established treatment options for IVF/ICSI-ET-related EPL.

## Future research priorities in IVF/ICSI-ET-related EPL

### Cell-specific mapping of immune dysregulation and pyroptosis at the maternal-fetal interface

A major priority for future research is to determine which cell populations at the maternal-fetal interface are most relevant to the interaction between immune dysregulation and pyroptosis in EPL following IVF/ICSI-ET. Although abnormal immune responses and inflammasome activation are biologically plausible contributors to pregnancy failure, it remains unclear whether pyroptotic signaling primarily affects trophoblasts, decidual macrophages, uterine natural killer cells, endometrial stromal cells, or multiple interacting cell types. High-resolution approaches, particularly single-cell transcriptomics and spatially resolved tissue profiling, may help define cell-specific inflammatory signatures and clarify whether pyroptosis is a driver, amplifier, or secondary consequence of local immune dysfunction.

### Temporal characterization of inflammasome activation during implantation and early gestation

In addition to cell specificity, the timing of inflammasome activation requires more precise investigation. Current literature provides insufficient information on whether pyroptosis-related signaling occurs before implantation, during the implantation window, or after early embryonic attachment and decidualization. This temporal uncertainty limits interpretation of causality. Future studies should therefore integrate temporally resolved sampling strategies in clinically relevant reproductive models to determine when NLRP3/ASC/Caspase-1 activation, gasdermin D cleavage, and IL-1β/IL-18 release occur in relation to implantation success or failure. Clarifying this sequence will be essential for distinguishing pathogenic mechanisms from downstream inflammatory epiphenomena.

### Validation of clinically relevant biomarkers and sample sources

Another urgent research priority is the validation of biomarkers that are both biologically informative and clinically feasible. Although several inflammasome- and pyroptosis-related molecules, including NLRP3, PYCARD, CASP1, IL-1β, and IL-18, have been proposed as candidate markers, their relevance to IVF/ICSI-ET-related EPL remains insufficiently defined. Future biomarker studies should compare tissue-based and circulating markers, assess specificity relative to other inflammatory reproductive conditions, and determine whether such markers add predictive value beyond established clinical variables. Standardized detection platforms, carefully phenotyped IVF/ICSI-ET cohorts, and independent external validation will be necessary before translational conclusions can be justified.

### Mechanistically grounded therapeutic translation

Therapeutic translation should proceed only after the biological framework has been more clearly established. Rather than broadly proposing immune modulators, artificial intelligence-based prediction systems, or nanotechnology-based delivery strategies in parallel, future work should first determine whether pyroptosis is causally involved in clinically meaningful subsets of IVF/ICSI-ET-related EPL. If so, subsequent studies may evaluate whether targeting upstream inflammatory priming, oxidative stress, or specific inflammasome components can improve reproductive outcomes without disrupting physiological maternal-fetal immune adaptation. In this context, translational progress will depend less on technological novelty alone than on rigorous mechanistic validation in human-relevant reproductive systems.

## Conclusion

EPL following IVF/ICSI-ET is a multifactorial reproductive outcome in which maternal age, embryonic factors, endometrial characteristics, and immune dysregulation all contribute to risk. Among these factors, disturbance of the maternal-fetal immune microenvironment is supported by growing evidence and likely represents an important component of implantation failure and early miscarriage. By contrast, although inflammasome-mediated pyroptosis provides a biologically plausible framework for inflammatory tissue injury, direct mechanistic and clinical evidence specifically linking pyroptosis to IVF/ICSI-ET-related EPL remains limited.

Accordingly, pyroptosis should currently be regarded as an emerging mechanistic candidate rather than an established central pathway in this setting. The available literature supports the possibility that immune imbalance, oxidative stress, inflammasome activation, and inflammatory cytokine release may interact at the maternal-fetal interface, but the strength, timing, and clinical relevance of these interactions remain to be defined. Therefore, any diagnostic or therapeutic implications related to pyroptosis should be interpreted cautiously and should not be overstated beyond the current evidence base.

Future progress in this field will require cell-specific, temporally resolved, and clinically grounded studies capable of distinguishing biological plausibility from causal relevance. A more precise understanding of immune-pyroptosis interactions may eventually improve risk stratification and identify new therapeutic opportunities in IVF/ICSI-ET-related EPL, but meaningful translation will depend on robust validation rather than extrapolation from adjacent inflammatory conditions.
